# Supplementary material for: Loss of Copine D Leads to Ras Activation in Dictyostelium discoideum
Source: Cells. 2026 Jul 31;15(15):1389. doi: 10.3390/cells15151389 (PMC13465345; doi:10.3390/cells15151389)
Supplement: Supplementary file 1 [file cells-15-01389-s001.zip › Supp Fig CpnD Manuscript.pdf]

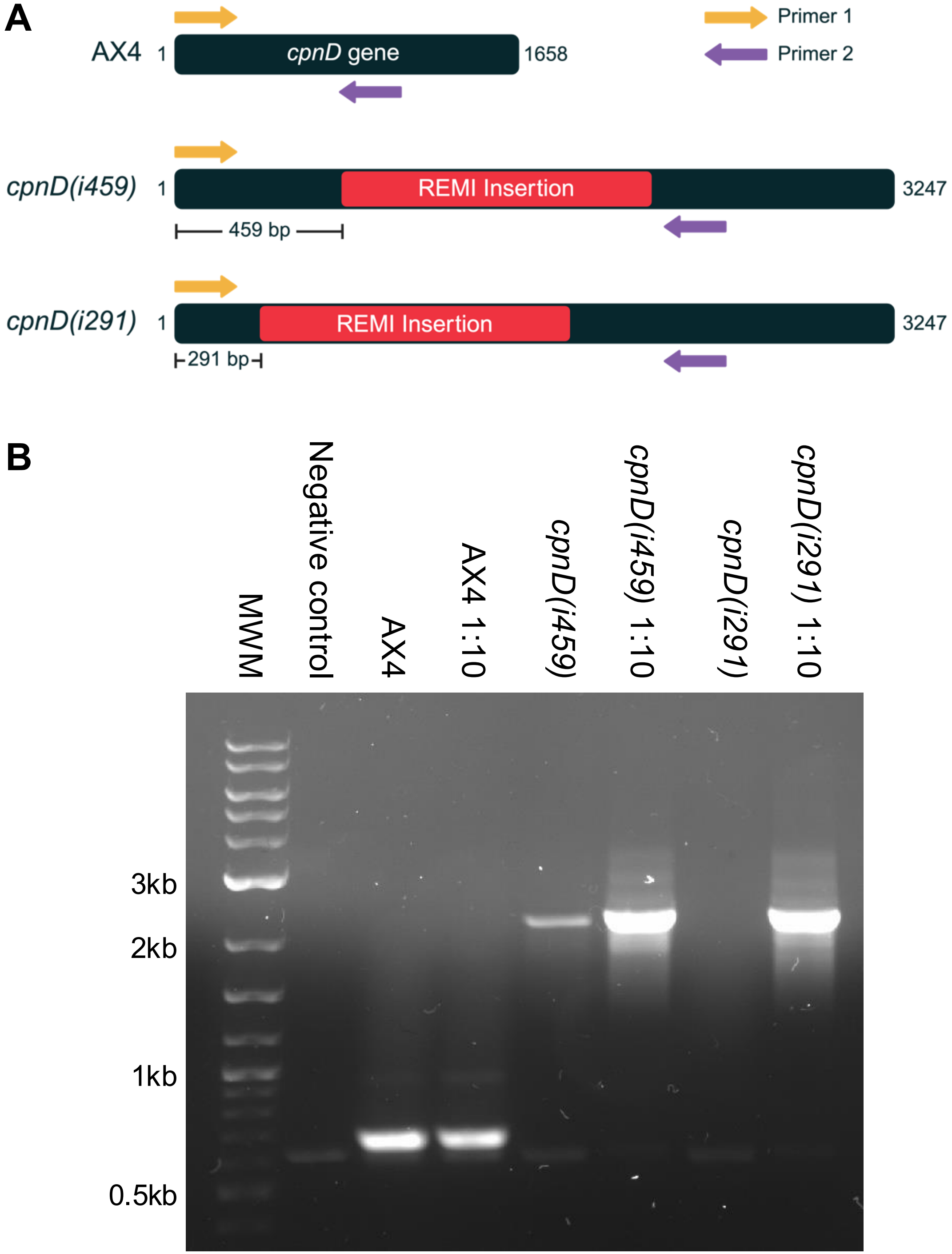
A
Primer 1
Primer 2
AX4
1
cpnD gene
1658
cpnD(i459)
1
REMI Insertion
3247
459 bp
cpnD(i291)
1
REMI Insertion
3247
291 bp
B
MWM
Negative control
AX4
AX4 1:10
cpnD(i459)
cpnD(i459) 1:10
cpnD(i291)
cpnD(i291) 1:10
3kb
2kb
1kb
0.5kb
