# Supplementary material for: Loss of Copine D Leads to Ras Activation in Dictyostelium discoideum
Source: Cells. 2026 Jul 31;15(15):1389. doi: 10.3390/cells15151389 (PMC13465345; doi:10.3390/cells15151389)
Supplement: Supplementary file 1 [file cells-15-01389-s001.zip › Supplemental Figure S1 Legend.pdf]

Supplemental Figure 1. PCR verification of REMI inserts in *cpnD* gene. A. Schematic showing the location of the REMI insert in each of the *cpnD* mutants and the location of the primers used in PCR. B. Genomic DNA was isolated from the parental AX4 cells and the two *cpnD* mutants and primers to regions of the *cpnD* gene on either side of the REMI inserts were used in PCR. PCR samples were analyzed by agarose gel electrophoresis and imaged.
